# Perceptual and Cognitive Factors Imposing “Speed Limits” on Reading Rate: A Study with the Rapid Serial Visual Presentation

**DOI:** 10.1371/journal.pone.0153786

**Published:** 2016-04-18

**Authors:** Silvia Primativo, Donatella Spinelli, Pierluigi Zoccolotti, Maria De Luca, Marialuisa Martelli

**Affiliations:** 1 IRCCS Fondazione Santa Lucia, Rome, Italy; 2 Dipartimento di Psicologia, Sapienza University of Rome, Rome, Italy; 3 Dementia Research Centre, UCL Institute of Neurology, University College London, London, United Kingdom; 4 University of Rome «Foro Italico», Rome, Italy; University of Leicester, UNITED KINGDOM

## Abstract

Adults read at high speed, but estimates of their reading rate vary greatly, i.e., from 100 to 1500 words per minute (wpm). This discrepancy is likely due to different recording methods and to the different perceptual and cognitive processes involved in specific test conditions. The present study investigated the origins of these notable differences in RSVP reading rate (RR). In six experiments we investigated the role of many different perceptual and cognitive variables. The presence of a mask caused a steep decline in reading rate, with an estimated masking cost of about 200 wpm. When the decoding process was isolated, RR approached values of 1200 wpm. When the number of stimuli exceeded the short-term memory span, RR decreased to 800 wpm. The semantic context contributed to reading speed only by a factor of 1.4. Finally, eye movements imposed an upper limit on RR (around 300 wpm). Overall, data indicate a speed limit of 300 wpm, which corresponds to the time needed for eye movement execution, i.e., the most time consuming mechanism. Results reconcile differences in reading rates reported by different laboratories and thus provide suggestions for targeting different components of reading rate.

## Introduction

Reading aloud is a complex task that involves several cognitive and sensory-motor subcomponents which range from feature detection to the comprehension of meaning and the pronunciation of words. Reading is a rich experience providing knowledge, emotions and in some cases a sense of beauty. This study does not address the full experience of reading nor it speaks about text comprehension. Rather, we focus on the perceptual, exploratory, memory and contextual components imposing an upper bound on reading speed. It takes many years to master this skill and during this long period of training each of the components improves substantially, showing specific learning effects. Literate adults read with almost perfect accuracy at an impressive speed, optimizing each of the processes and performing them in parallel. Carver (1992) [1] proposed that with long practice reading approaches an optimal rate of about 300 words per minute (wpm) and simultaneously enables orthographic decoding, pronunciation and comprehension of meaning. The most efficient comprehension was obtained at the same optimal rate of 300 wpm for both reading and listening [2]. Carver created the term “*rauding”* [1] to indicate the processing by which reading and listening (or rauding) lead to the ability to understand what has been read. According to Carver, readers may slow down or speed up their reading rate at the expense of some of the cognitive components involved in achieving different goals. If the reader’s aim is to learn, the rate does not exceed 200 wpm, and will be even slower if memorizing is required (about 140 wpm). Vice versa, higher rates may be achieved if the task requires skimming to find transposed words in a sentence (i.e., “*Horse the trotted toward*” instead of “*The horse trotted toward*”), with readers reaching a speed of 450 wpm. Furthermore, when the goal is to find a target word in a text (visual scanning), the rate increases up to 600 wpm [1]. Thus, reading rate adapts to the goals of the observer; however, most of the time individuals read at the rauding rate [3].

The aim of the present study is to measure the upper limits of reading rate beyond the limits imposed by comprehension. Rapid Serial Visual Presentation (RSVP), first introduced in 1970 by Forster [4], is a method to study the rate of processing in reading. However, as discussed later, it provides extremely variable estimates of reading speeds. A second aim of this study is to account for differences across labs and conditions in the reported reading rates.

Reading rate depends on multiple components. The processing time needed to read aloud a word or a sentence (without any special memorizing or learning requirement) may be roughly ascribed to three subcomponents, i.e., eye movement execution, decoding and speech production. In standard conditions all of these subcomponents have an effect on reading rate. The most time consuming process, i.e., pronunciation, presumably dominates over decoding and eye movements, setting this speed limit. Recently, the idea by Buswell’s (1921) [5] of eye-voice span has been revived after some years in which this measure was not used extensively (as eye movements studies focussed on silent reading) [6,7]. This helped to separately describe the subcomponents involved in reading aloud; in particular, it appears that the distance in time between decoding (as assessed by eye fixation position on a line of text) and pronunciation varies with reading skill and represents an idiosyncratic trait of the reader [5]. This is in keeping with Carver’s suggestion that reading speed is a stable trait of task execution. Thus, readers slow down the exploratory pattern by delaying the decoding of new material to wait for pronunciation, which sets an upper limit for speed. This is one of the relevant elements suggesting to select a measure of reading that excludes pronunciation, as we did in the present study.

The prevalent method used to minimize the role of pronunciation time is to examine vocal reaction times (RTs), i.e., the time between stimulus onset and the onset of the observer’s vocal response. Indeed, much of the literature assessing orthographic processing and constructing cognitive models of word recognition is based on vocal RTs (e.g., connectionist dual process CDP++ [8]). Not surprisingly, vocal RTs for single words produce very different speed estimates relative to standard text reading; however, this difference is in the opposite direction to that expected. Adult readers’ vocal RTs to single short word (i.e., excluding word utterances) is about 400 ms, which gives an estimated reading speed of 150 wpm, which is much slower than the 300 wpm obtained with functional text reading [1]. How can this difference be explained? Basically, multiple stimulus arrays (such as standard texts) enable the parallel execution of the reading sub-components with associated time benefits. Indeed, a clear advantage of multiple word arrays compared to single word presentations can be demonstrated if an appropriate measure (total reading time per item) is used [9]. Moreover, a study that modelled speed of reading based on vocal RTs showed that this variable is not an ideal measure for estimating reading speed because it is dominated by the time-consuming sensory-motor sub-component [10]. By contrast, RTs prove very effective in studying small lexical effects (i.e., in the order of a few tenths of ms).

Overall, two factors weight heavily on the measured reading rate: pronunciation on one hand (as in reading aloud studies) and limited requirement for parallel processing on the other (as in most RTs studies). RSVP is one method that excludes the pronunciation times (unlike reading aloud), and at the same time allows for the processing of multiple items (unlike single word vocal RTs). In this procedure one word at a time is briefly presented, followed by another one, in the same spatial position (4 to 8 words are typically presented in a single stream). Reading rate in RSVP is typically defined as the word presentation rate at which an individual reads at a criterion level of accuracy. As for all threshold measurements this enables comparisons across conditions at the same level of task difficulty. Compared to standard reading rate measurements RSVP usually generates much higher reading rates. Indeed, Potter [11] showed that reading and recall was still excellent at 12 words per second (i.e., 720 wpm). However, it has also been suggested that such high rates are suboptimal for text comprehension [12,13]. Estimates of reading up to 700–1000 wpm are frequent [14,15]. Two factors might be responsible for RSVP speed. First, the role of eye movements is minimized by presenting stimuli at the same spatial location. Second, the articulatory component does not contribute to the estimation of reading rate. Thus, RSVP provides speed estimates that are independent from these components. Finally, reading under RSVP resembles functional reading more closely because, similar to functional reading and differently from RTs paradigms (where processes are forced to be serial) it allows parallel processing of several words. Due to the short presentation time of each word and the absence of a temporal interval between successive words, readers are forced to encode the preceding word while they decode the letters of the following word [14].

### Variability in RSVP reading rate estimates across laboratories

RSVP has been widely used to study many low level aspects of visual processing e.g., in [16,17]. As discussed above, although we should expect reading rates measured by RSVP to be very similar across studies, different studies that used the same paradigm found quite different reading rates. In some cases the advantage provided by the RSVP technique in speeding up reading rates was relatively small, with mean reading rates of ca. 300 wpm [16,17,18,19,20]; in other studies, reading rates exceeding 1500 wpm were reported [14,20]. We set out to discover the origin of these discrepancies.

Our working hypothesis was that methodological differences among studies using the RSVP paradigm were responsible for the different outcomes. The aim of the present study was to understand the weight, in terms of processing time, of the various components that contribute to the reading process in the RSVP procedure. For this purpose we designed various experiments manipulating the relevant variables of the procedure and measuring their relative contribution. Although this study was specifically tailored to test variables using the RSVP method, it might also contribute to the more general goal of clarifying the sources of contrast in the literature concerning reading rate.

In the following sections we review the RSVP literature on reading rate by considering the factors that are responsible for the performance in this task and may differentially contribute in generating the described contrasting results. Based on these considerations, we propose experiments designed to tease out the role of each of these factors. In particular the focus here is on the decoding processes. Text comprehension was not evaluated since it goes beyond the scope of the present work.

### Effect of sequence and cross-item masking in RSVP

Reading rate is estimated according to the duration threshold necessary to reach a given level of accuracy in reporting the stimuli (typically 80%). Notably, this threshold is calculated by considering the total number of words reported correctly, without distinguishing between the ordinal positions of each word in the sequence stream. However, words in the stream are not all recognized at the same level and the first and last words may have an advantage. Pelli & Tilman (2007) [21] noted that “*in order to minimize end-effects in the 6-word sequence of a trial*, *we added a random letter string before the first word in a trial and another after the last word […] Without temporal flankers*, *the first and last words in a trial showed a strong advantage over the middle four*. *With the temporal flankers*, *there is no longer any advantage for the last word in a trial*. *The primacy effect*, *higher report of the first word with respect to the following words*, *was reduced*, *but not eliminated*, *by the addition of temporal flankers*”. The authors refer to the visual masking phenomenon, i.e., to the fact that a stimulus is reported less accurately if it is presented close in time to other stimuli, with the first one masking the following one [22,23,24]. Thus, we hypothesize that this methodological difference may be responsible for some of the discrepancies in reading rates reported in the literature. It should be kept in mind, however, that the advantage for the first and last words was mentioned only by Pelli and Tilman [21] and the phenomenon has never been studied in detail. Therefore, in Experiments 1 to 3 we evaluated the speed of reading at threshold as a function of the word position in the stream and of the presence/absence of visual masks.

### Eye movements in RSVP

The RSVP paradigm was originally developed to minimize the weight of the eye movements that are necessary when reading a text distributed across the whole screen or page [11,25,26]. Rubin and Turano [14] compared reading rates in RSVP and in static texts (where 200 or more words were presented simultaneously on the screen and reading rate was calculated as the number of words correctly read aloud in a minute). These authors obtained higher reading rates for RSVP (about 1000 wpm) than for static text (about 300 wpm) and concluded that programming and executing eye movements imposes an upper limit on reading rate. Unlike RSVP, standard text reading requires the execution of eye movements; however, it also requires pronunciation, which adds a large and relatively constant time to the overall response [6,10]. Thus, the measured speed difference found by Rubin and Turano [14] may overestimate the role of eye movements. In the present study we quantified the weight of eye movements and measured the impact of a classical RSVP presentation of the stimuli in terms of reading rate speeding up as compared to a distributed presentation of the words (Experiment 4).

### Semantic context in RSVP

Most psychophysical studies of reading rate based on RSVP are concerned with the visual properties of the stimuli and generally ignore the lexical and semantic status of the words. However, top-down semantic factors may affect reading speed because the presence of context greatly accelerates sentence reading [18]. Consequently, differences in estimated reading rates across studies may also be due to this often uncontrolled source. So far studies using the RSVP paradigm have not been consistent with regard to the type of materials used for reading: sentences, scrambled text and lists of unrelated, random words have often been used interchangeably.

In 2007 Pelli and colleagues [17] studied differences in reading rate when ordered or scrambled sentences were presented. Scrambled sentences reduced the variability in the rate measure. The effect of the context gain (i.e., the increased reading speed for ordered vs. scrambled sentences) has been investigated in depth [17,19,27,28,29,30]. To our knowledge, however, the reading rate gain for sentences (ordered or scrambled) compared to lists of random words has not yet been investigated systematically equating for task difficulty across conditions (i.e. by the use of threshold). Thus, in Experiments 5 and 6 we investigated the effect of semantic context on reading rate by contrasting low-level factors, such as visual masking, with high-level factors, such as memory limitations.

### Role of working memory in RSVP

In experiment 6 we studied the role of working memory in association with semantic context. In the RSVP literature there is great variability regarding the number of stimuli displayed in each trial and there is no definite information about the effects of these variables on reading rate. It is likely that this display variability account for difference between reading speed measured in different laboratories. The number of items in the stream varies considerably: In some studies e.g., [19,20,21] it is in the short-term memory span [31], with estimates of speed ranging from 300 to 1300 wpm (median: 590). In other studies the number of stimuli in the stream exceeded the memory span e.g., [16,17,32,33,34,35,36,37] obtaining rates ranging from 250 to 800 wpm (median: 419). These outcomes are not directly comparable, however, because many other uncontrolled factors besides stream numerosity are involved (e.g., stimulus type, presence/absence of masks, threshold criterion, etc.). A direct comparison between conditions characterized only by the different number of items per trial (carried out in Experiment 6) will help explain these discrepancies.

## General method

Unless stated otherwise the same general method was used for all the experiments described below.

### Stimuli

Stimuli were either lists of unrelated words or meaningful texts. Words were selected from the LEXVAR database (http://www.istc.cnr.it/grouppage/lexvar) [38]. Lists of words within each experiment were matched for word frequency and bigram frequency. Texts were derived from the Italian translation of “Alice in Wonderland” by Lewis Carroll; in some cases, lists of unrelated words from this book served as control stimuli (see below). In all cases words were presented in Courier New font, which is proportionally spaced. Each letter subtended 0.4 deg of visual angle and the centre-to-centre distance between adjacent letters was 0.47 deg. Text was presented in high contrast: black letters on a white background.

### Participants

Different subjects took part in different experiments of the study; all had normal or corrected to normal vision. They were all Italian native speakers and did not report developmental reading disorders. All the subjects gave written consent to participate in the experiments. This study has been approved by the Ethics Committee of the IRCCS Fondazione Santa Lucia Rome (Prot. CE-PROG.480).

### Procedure

Participants were seated 57 cm away from a 15.5 Sony Vaio laptop (refresh rate = 60 Hz). A fixation point (a black square subtending 0.2 deg of visual angle) was presented at the center of the screen for 200 msec. Immediately after offset of the fixation point 4 words were presented using the RSVP paradigm, i.e., words were presented sequentially, one word at a time, at the same location on the display. There was no blank frame (zero inter-stimulus interval) between words. The presentation duration varied as described below. When a mask was used (######) it was presented immediately before the first word and immediately after the fourth word. The mask had the same stimulus size as the words in the stream. The mask also had the same stimulus duration which varied in each trial according to the participant’s performance. As for the stimuli, the mask’s symbols were black on a white background.

In all experiments participants were asked to read aloud the words presented in the stream, while the words exposure duration varied across trials (see below). Participants were instructed to read the words as accurately as possible. They were informed that they could take their time to report the words in any order also after the end of the trial sequence. Participants were alerted that some of the trials would seem easy because the words remain for long time on the screen, and some would appear hard or impossible because of the brief presentation time. In each trial the number of reading errors were counted. Productions that did not correspond to the presented word, as well as word omissions were considered error. In each trial sequence, each word was considered as an independent estimate of accuracy for the particular exposure duration tested.

We measured the duration threshold for each participant and each condition by varying exposure duration in a 40-trial run using the improved QUEST staircase procedure with a threshold criterion of 80% correct responses [39]. The adaptive QUEST procedure increased or decreased the presentation rate (starting from 500 ms) according to the participant’s accuracy. Separate thresholds for each tested condition were measured through independent runs. The order of the tested conditions (e.g., masked vs. unmasked condition in Experiment 1) was counterbalanced across participants. The word order of presentation in each run was fully randomized. When the duration was near the threshold (or at threshold) utterance of the response always started after the offset of the word sequence (i.e., words flashing rate at or near threshold is always below vRTs of adult fluent readers). In experiments 1 to 5 the word sequence in each trial was short so not to exceed the memory limitations (i.e. 4 words in trial). In experiment 6 the effect of memory limit on RSVP reading speed was measured by varying the number of words presented in a trial (i.e. 6 or 12). In all cases there was no time limit for completing the response.

In experiments 1 to 4 the stimuli were selected from balanced lists of random words (more details in the specific method sections). As an example the participant could be shown with “calore” (heat), “altare” (altar), “febbre” (fever) and “lancia” (spear) in one trial. To evaluate the effect of context in experiment 5 and 6 sentences were also used, and in a trial the words “la sorella stava leggendo” (the sister was reading) could appear in sequence.

### Data analysis

Reading rate (*i*.*e*., words per minute, wpm) was measured as 60/duration threshold*1000. In all experiments log-transformed values of reading rate were entered in the statistical analyses. In all experiments the effects of experimental manipulations were compared by means of ANOVAs (factors are presented in the single analyses). Fisher's least significant difference (LSD) post-hoc tests were used whenever appropriate.

## Experiment 1: Effect of Masks on Reading Rate in RSVP

Studies using the RSVP procedure to investigate reading speed can be roughly divided between those that used a mask e.g., [18,19,21,32,33,34,35] and those that did not e.g., [14,16,20,36,37,40]. Notably, this factor is rarely considered in the interpretation of results. We propose that the presence/absence of a mask may have substantially contributed to the different outcomes obtained.

In Experiment 1, the role of the mask in reading is quantified by comparing a condition in which observers are asked to read aloud a stream of four words, preceded and followed by a mask (a string of identical symbols) with a condition in which no masks are used.

### Method

Two lists of 160, 6-letter words matched for frequency (mean frequency = 50.6 and 50.2 for list 1 and list 2 respectively; p > .1) and bigram frequency (11.2 and 11.7, respectively; p > .1) were used. Nine subjects participated in the study. Their mean age was 25.5 years (S.D. = 2.8; range = 21–30).

Two conditions were tested: on the first one no mask was used while on the second one a mask was used as described in the General Method section.

### Results and comments

**Fig 1:**
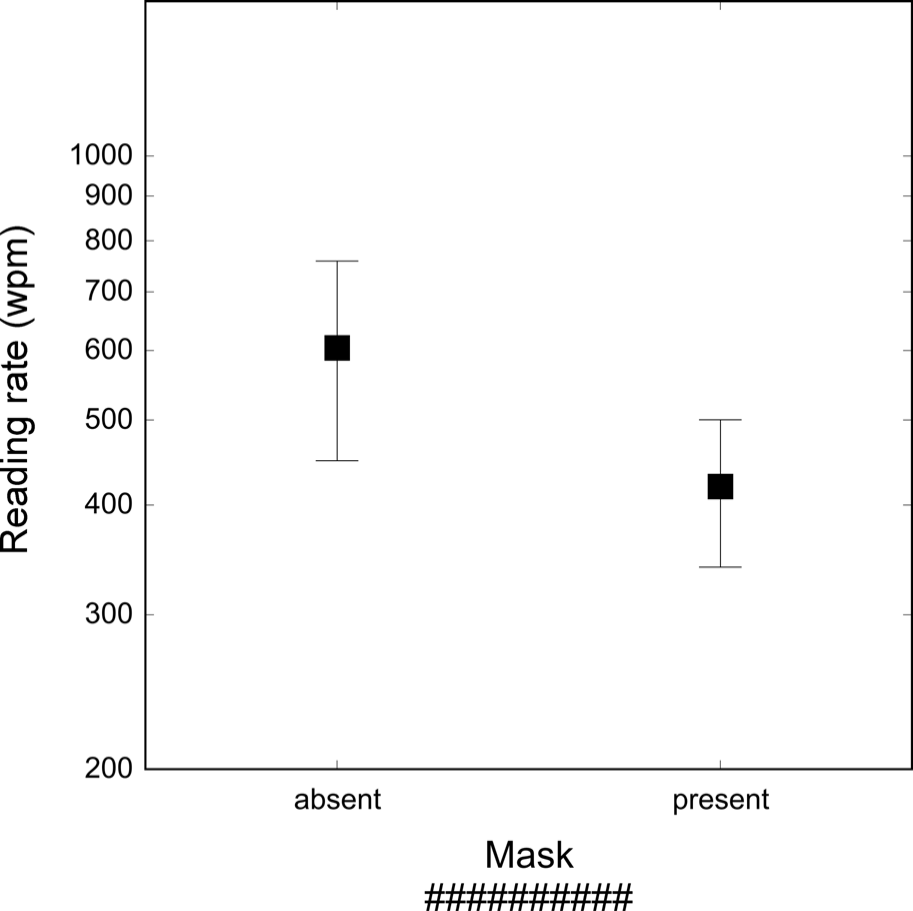
Reading rate (wpm) in the absence (left) and in the presence (right) of a mask. The features of the mask are shown above. Bars represent standard deviations.

Reading rates for words presented in the mask and no mask conditions are presented in Fig 1. The presence of the mask reduced reading speed from 604 to 420 wpm, i.e., by a factor of 1.5 (F_1, 8_ = 12.34, p < .01).

Results indicate that the presence of a mask before the first and after the last word of the stream causes a steep decline in reading rate, with an estimated cost of around 200 wpm. When using the mask the first and last words of the stream become perceptually more comparable to the other words in the stream (i.e., they lose their advantage). Indeed, the second and third words of the stream are preceded and followed by words and these words may act as masks, making the target word less visible.

The decline in reading rate with masks indicates that the detectability of the first and last words in the stream plays an important role in the overall evaluation of reading rate. Thus, it is likely that words that have different position in the sequence contribute with a different weight to the reading rate measured by RSVP. We address this issue in the second experiment.

## Experiment 2: Effect of the Position of the Word in the RSVP Stream Sequence (Without Masks)

In Experiment 2, we investigated the weight of each word in determining the duration threshold within the four-word stream. To this aim, although keeping constant the general method procedure, we measured the duration threshold separately for each ordinal position in the sequence (i.e., either the first, second, third or fourth position). Note that this procedure differs from standard measurement, where the threshold is calculated by averaging the report for all words, regardless of their position. The participants’ experience was however the same. In this experiment we did not use the mask before the first and after the last word.

### Method

We used four lists of 120 stimuli that were matched for frequency (mean frequency = 54.5, 54.7, 54.5 and 54.3, all p_s_ > .1, respectively) and bigram frequency (11.22, 11.20, 11.23, and 11.25, all p_s_ > .1, respectively). Stimuli were 4 and 6 letters long (N = 48 and 72, for each list, respectively).

Six subjects participated in the study. Their mean age was 25.7 years (S.D. = 3.7; range = 19–28). Equipment and general procedure were the same as those of Experiment 1 except for the following: a) no mask was used; and b) the duration threshold estimate was evaluated separately for the four ordinal position of the words in the stream (in four separate runs). Participants’ experience was that they were required to take part in 4 identical experiments (only the words lists were different but matched; see above). However, in each of the 4 runs we adapted the stimulus duration on the basis of the participant’s performance only on the first, second, third or fourth word in the stream, respectively. Accordingly, for each specific run, the reading rate threshold was measured for the first, second, third or fourth word, respectively.

Thresholds were measured in 30-trial runs. Observers were naïve as to the order of the word in the stream considered in the threshold measure and had to read all four words in the stream. Regardless of which word in the sequence was considered in the adaptive procedure, the stimulus duration was consistently varied for all four items.

### Results and comments

**Fig 2:**
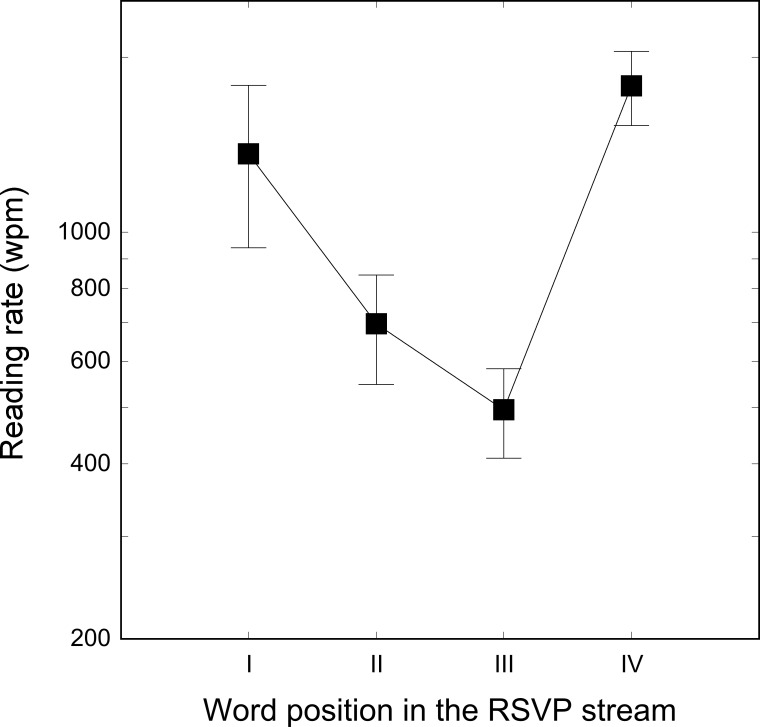
Reading rate measured separately for each of the four words in the stream. Each threshold was independently measured with a 120 words list, for a total of 480 stimuli in the experiment. No mask was used. Bars represent standard deviations.

A repeated measures ANOVA was run with word ordinal position as repeated factor. The main effect of word ordinal position was significant (F_3, 15_ = 54.44, p < .0001). As shown in Fig 2, the first and last words had the highest reading rates (1365 and 1787 wpm, respectively). LSD post hoc comparisons revealed that the first word had a higher reading rate than the second (696 wpm, p < .0001) and third (496 wpm, p < .0001) words. Similarly, the fourth word in the stream had a higher reading rate than the other three words in the stream (all p_s_ < .05). The difference between the second and the third word was also statistically significant (p < .01), with a higher reading rate for the second word.

Results indicate that the four words in the RSVP stream are not equally visible but the first and last words are much more easily reported. Notably, in standard RSVP measurements, reading rate is estimated by taking into account accuracy on all words independently of their position in the stream.

In keeping with the results of Experiment 1, we propose that words in central positions in the sequence suffer from the visual masking generated by preceding and following words. By contrast, the absence of a non-verbal mask before the first word and after the last one increases reading rate by a factor of four, which allows reaching an impressive reading speed independently of the presence of a word to be processed after (as for the first word in the sequence) or before (as for the last word in the sequence) the target word. The estimated consequence of having unmasked signals amounts to a difference of about 1000 wpm between items. Taken together the results of Experiments 1 and 2 show that reading rate is slowed down when two masks (one preceding and one following the target word) are used and suggest that this masking effect may be independent from the nature of the mask (non-verbal vs word). The aim of Experiment 3 was to test this hypothesis by measuring the letter masking cost on the third and fourth words in the stream netted from the first and last unmasked word advantage.

## Experiment 3: Effect of the Position in the RSVP Stream Sequence (With Masks)

To further investigate the role of the non-verbal mask in modulating reading rate, here we added the masks and measured the duration threshold separately for each ordinal position in the stream. All stimuli and conditions were the same with the exception that we introduced a mask (######) immediately before the first word of the stream and immediately after the last word.

### Method

We used the same lists of words as in Experiment 2. Ten subjects took part in the study. Their mean age was 26.7 (S.D. = 1.64; range = 25–29).

The general method described above was followed. A mask immediately preceded and followed the first and last words of the stream.

### Results and comments

**Fig 3:**
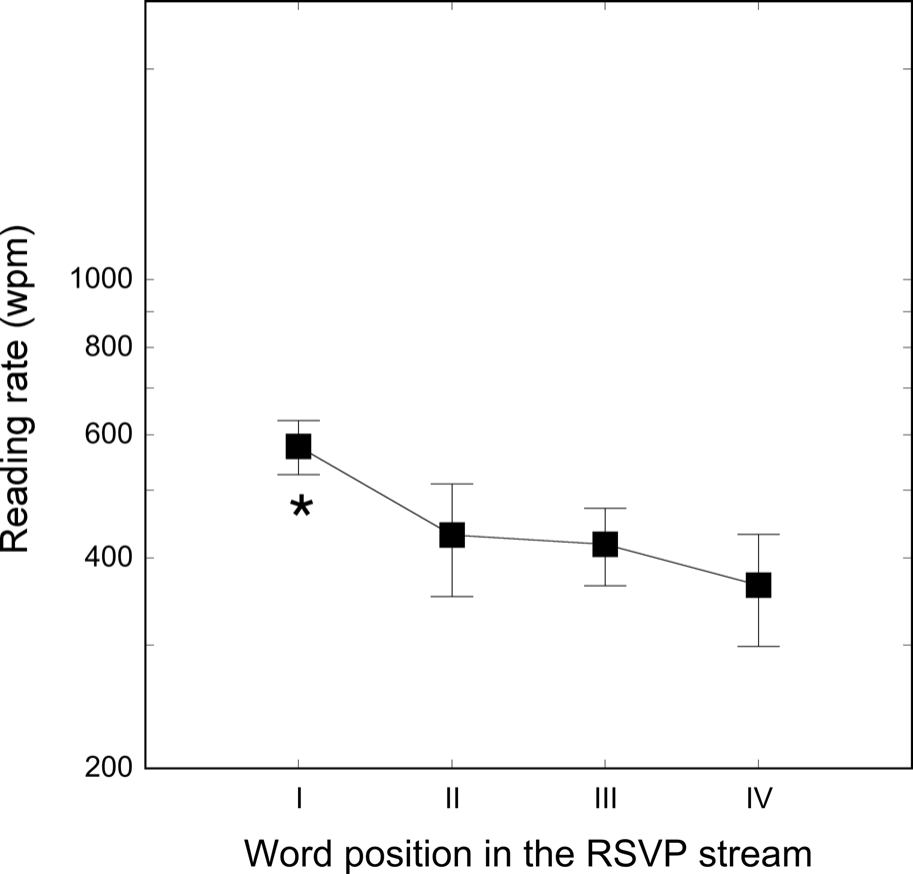
Reading rates separately for each of the four word ordinal positions in the stream. As in experiment 2 each threshold was independently measured using a 120 words list. The first word was immediately preceded, and the last word was immediately followed, by a mask. Bars represent standard deviations. * indicates p-value < .01.

Results are shown in Fig 3. The main effect of word ordinal position was significant (F_3, 27_ = 9.92, p < .001). Post-hoc comparisons indicated that the first word had a higher reading rate (578 wpm) than the second, third and fourth words in the stream (431, 418 and 365 wpm, respectively; all ps < .01), which did not differ from each other (all p_s_ > .1).

The results of Experiment 3 confirm that adding a mask immediately before and after the stream of words makes the words in the stream more similar. Taken together these three experiments a) indicate that reading speed is higher when no masks are used, and b) to compare reading rate across different labs, the presence/absence of non-verbal mask at the beginning and the end of the sequence should be taken into account.

Although substantially reduced by the presence of the preceding mask up to a difference of 800 wpm, the readability of the first word was still about 150 wpm faster than that of the other words. In the masked repetition priming paradigm interactions may occur at different processing levels, from feature analysis to lexical entry encoding, and may involve different brain regions e.g., [41]. A non-verbal mask (such as that used here) may be seen as a weak mask relative to a word that shares both (short segments of various orientations) and elementary units (letters) with the target. However, this interpretation appears unlikely as the advantage for recognizing the fourth word in the stream by eliminating the non-verbal mask was quite large, i.e., about 900 wpm (compare data of Figs 2 and 3). Alternatively, the advantage of the first word over the last may indicate a reliable, although small (a factor of 2 or less), effect in accuracy determined by primacy in memory recall e.g., [42,43].

Overall, the effect of masking between consecutive words presented in the same spatial position was large, ranging from 600 to 1000 wpm in the unmasked condition. The similar effect of orthographic and non-orthographic material on word reading thresholds indicates that masking occurs at an early stage of processing (either features or letters) before the words are lexically encoded. This interesting finding will be considered in greater depth in the general discussion.

## Experiment 4: The Cost of Eye Movements on Reading Speed Measured with RSVP

Reading speed is greatly enhanced by RSVP due to the reduced involvement for eye movements [14]. In this experiment, we aimed to quantify the role of eye movements by comparing a condition in which the four words are presented in the same spatial position (i.e., eye movements are minimized) with one in which words are simultaneously presented along the horizontal meridian and subjects have to move their eyes to read them. This comparison allows for assessing eye movement costs, net of the influence of pronunciation time, because in both conditions the utterance of the response at threshold always starts after the offset of the stimulus. Thus, this comparison should facilitate assessment of the eye-movement cost on reading speed.

### Method

Two lists of 120 stimuli matched for frequency (mean frequency = 54.5 and 54.7 respectively, p > .1) and bigram frequency (11.22 and 11.20 respectively, p > .1) were used. Stimuli were 4 and 6 letters long (N = 48 and 72, respectively, for each list).

Eight subjects participated in the study. Their mean age was 23.9 years (S.D. = 2.03; range = 21–27).

Two conditions were used. In the first condition words were consecutively presented in the same spatial position at the center of the visual field; in the second condition words were simultaneously presented along the horizontal axis, i.e., they were arranged horizontally across the entire screen (subtending 34 deg). Specifically, 4-letter words subtended 4.5 deg and 6-letter words subtended 7 deg. The horizontal distance between words varied accordingly: 4 deg (distance between the two 4-letter words); 2.5 deg (distance between the 6-letter and 4-letter words); 1.5 deg (distance between two 6-letter words). In the second condition of the experiment, eye movements were required in order to fixate the words presented on the screen.

### Results and comments

**Fig 4:**
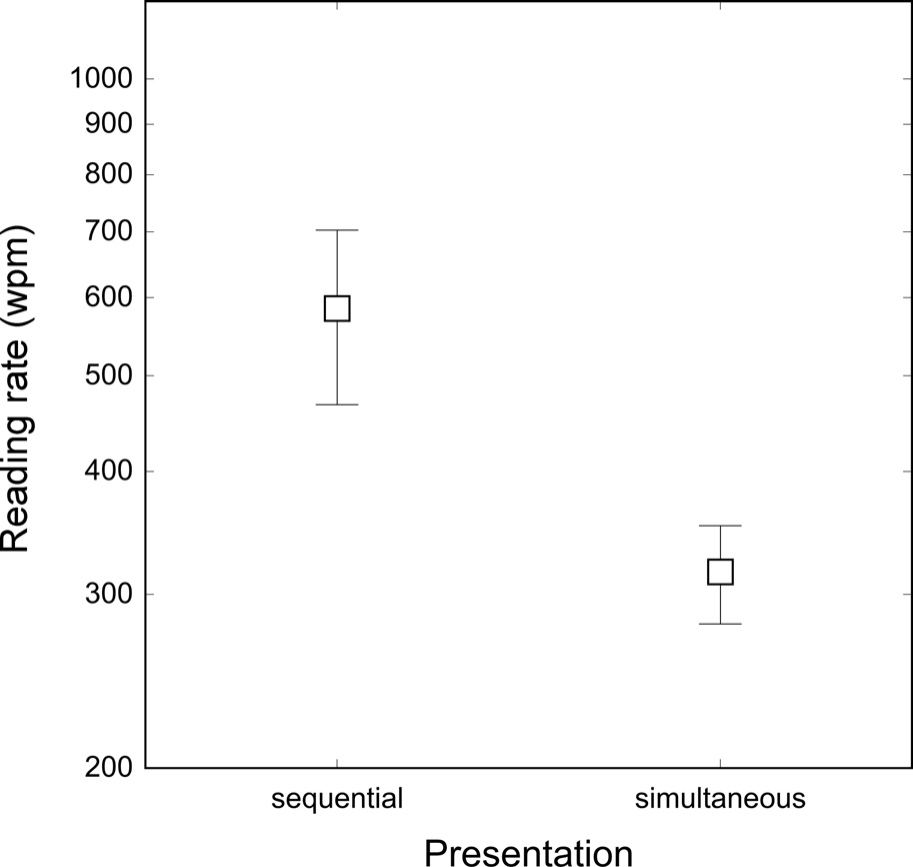
Reading rate (wpm) for words in the sequential and simultaneous presentation conditions. No mask was used. Bars represent standard deviations.

As shown in Fig 4, reading rate was much lower (316 wpm) with the horizontal layout of the stimuli (requiring eye movement scanning) than with the same spatial position presentation (585 wpm), where eye movements were not required (F_1, 7_ = 8.88, p < .05).

By using streams of words presented in the same spatial location, thus limiting the need for eye movement scanning, the RSVP procedure effectively maximizes reading rate. In fact, when eye movements are made necessary to read comparable words (i.e., sequential condition) the reading rate drops considerably. It must be noted that although the estimated cost of eye movement execution is large, ca. 250 wpm, it is much smaller than the 900 wpm advantage reported by Rubin and Turano [14] for RSVP reading. Although our simultaneous condition produced a speed similar to their 200-word static text (about 300 wpm), the RSVP condition failed to produce the high rate of 1200 wpm reported by these authors. We believe that this discrepancy may be due to the weaker letter-masking effect in Rubin and Turano study [14] when short and long words were presented in sequence (which typically occurs in the case of standard text). We will return to this issue in Experiments 5, 6 and 7.

The results of the experiment show that minimizing the need for eye movements can have a 250 wpm reading rate advantage. Eye movements have a cost that sets the upper limit of reading rate to about 300 wpm. It is however worth noting that the time costs associated with eye movements in the present condition might have been overestimated, since the distance between words was larger than what normally present in text reading.

## Experiment 5: Effect of Context on RSVP Reading Rate

Context is an important factor in modulating reading rate in foveal reading [16,17,18,19,20,27]. The gain in reading sentences as compared to scrambled words from the same sentences is usually greater than 1. However, Experiments 1 to 3 showed that another factor is quite important in modulating reading speed, i.e., the masking between successive words presented in the same spatial position. One interesting possibility is that the very fast rate for text reading is not entirely due to the facilitating effect of context. When sentences are presented with the RSVP procedure successive words in the stream often have different lengths. Different from the case in which lists of words of the same length are presented, length differences of a sentence should reduce masking between consecutive words. To disentangle the role of context from that of masking between letters we selected three types of stimuli: ordered text, scrambled text and random words of a length comparable to the average number of letters of the nouns in the text (i.e., 4 or 6 letters).

### Method

In the ordered text condition, the first 160 words of the first chapter of “Alice *in Wonderland*” were presented in the exact order in which they appeared in the text (mean length of the words was 4.85 letters, S.D. = 2.9; range 1 to 15). In the scrambled condition, the first 160 words in the second chapter of the book were randomly presented in the streams of words (mean length of the words = 4.94 letters, S.D. = 3.1; range 1 to 18). In both conditions, punctuation was eliminated and no capital letters were used. In the third condition a list of 160 words was generated from the LEXVAR database [38]. Stimuli were 4- and 6-letter words (N = 48 and 72, respectively, for each list). The three stimulus types were presented to different subjects with and without a non-verbal mask (displayed at the beginning and the end of each word stream). We followed the general method described above manipulating, in two different conditions, the presence or absence of the mask.

Six subjects (mean age = 27.3; range = 25–30, S.D. = 2.06) participated in the first unmasked condition (ordered text, unordered text and random words-no mask). Six other subjects (mean age = 27.2 years; range = 25–30, S.D. = 1.92) participated to the second masked condition (ordered text, unordered text and random words—non-verbal mask).

### Results and comments

**Fig 5:**
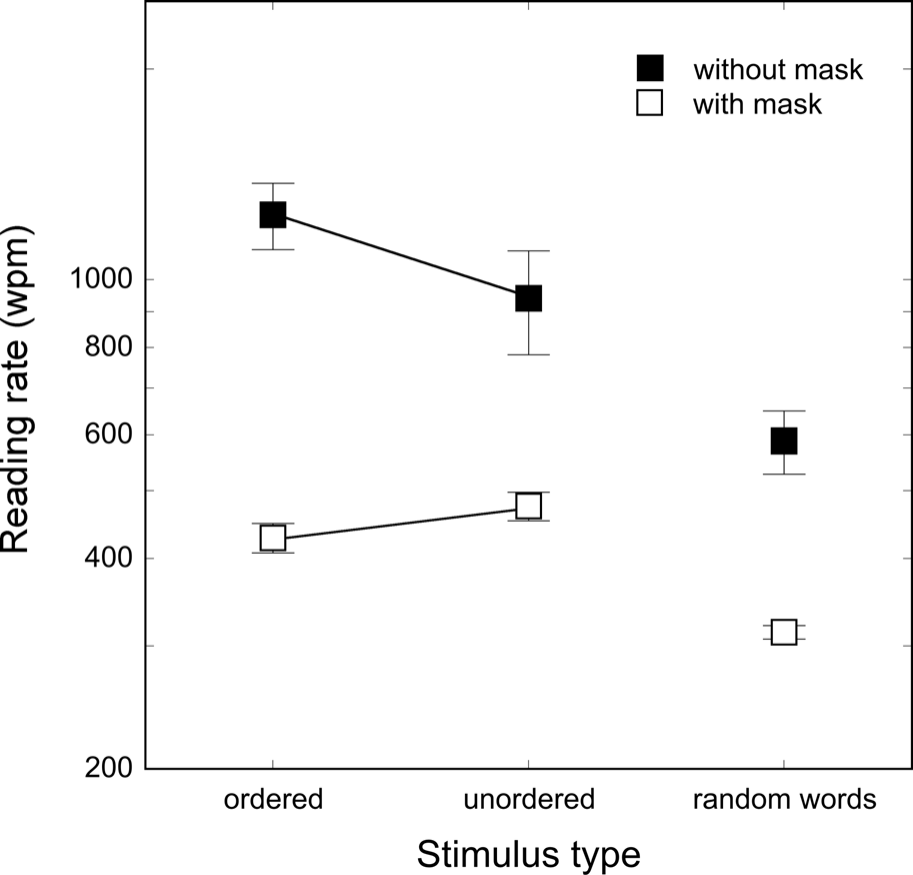
Reading rate for ordered text, unordered text and 4–6-letter random words. Bars represent standard deviations. Data were collected in two groups of subjects in the absence (black squares) or in the presence (white squares) of a non-verbal mask before the first word and after the last word in the stream.

Results are shown in Fig 5; black squares indicate the no mask condition and white squares indicate the masked condition.

The ANOVA for the no mask condition indicated a significant main effect of stimulus type (F_2,10_ = 13.21, p < .002). P*ost-hoc* comparisons showed that although ordered text was read faster (1239 wpm) than scrambled text (940 wpm) this difference did not reach significance (p = .09). Significant differences emerged when comparing random words (588 wpm) with both ordered (p < 0.001) and scrambled text (p < 0.01). In the masked condition, a significant main effect of stimulus type emerged (F_2, 10_ = 40.07, p < .0001). *Post-hoc* comparisons indicated a marginally significant difference between ordered and scrambled text (428 and 475 wpm, respectively; p = .055) with the latter read faster than the former. Both ordered and scrambled sentences were read faster than random words (314 wpm; both p_s_ < 0.0001).

An inspection of Fig 5 shows a large difference between the conditions with and without a mask. As in Experiment 1, the presence of a mask led to a drastic reduction in reading speed in all conditions. To compare the conditions with and without masks we ran a 2 x 3 ANOVA with mask (yes, no) as between subjects factor and stimulus type (ordered text, scrambled text, random words) as repeated factor. The analysis indicated the significant main effects of the stimulus type (F_2, 20_ = 26.98, p < .00001) and mask (F_1, 10_ = 93.37, p < .00001) factors. The stimulus type by mask interaction was significant (F_2, 20_ = 4.71, p < .05): in the no mask condition significant differences emerged among all conditions while, in the mask condition, only the differences between ordered text and random words and between scrambled text and random words were significant (p < .05 and .01, respectively). The difference between ordered and scrambled text was not significant (p = .35).

The effect of the additional mask (before the first and after the last word) was associated with considerably lower reading rate, which confirms previous results.

In the case of the unmasked condition, we obtained a reading rate comparable to that reported for English texts [14]. The faster reading rate obtained with both ordered and scrambled text as compared to the lists of words of similar length is in keeping with our proposal that masking between letters of consecutive words is an important factor in reducing reading speed. In fact, with both ordered and scrambled sentences, words of quite variable length (ranging from 1 to 18 letters) followed one another. Presumably, this length difference limited the masking effect between letters. By contrast, masking between letters of consecutive words was maximized in the random word condition where 4- and 6-letter words were presented. Notably, the effect of letter masking was much more evident than the effect of semantic context (comparison between ordered and scrambled conditions), which was small and had a variable direction.

Nevertheless, various criticisms can be raised regarding the interpretation of these findings: the random words were not drawn from the same linguistic context as those in the ordered and scrambled conditions. As a consequence, 1) random words could have several different linguistic features (in particular they could have a lower frequency) with respect to words of ordered/scrambled sentences and this could be the cause for lower reading rate; 2) random words included only nouns, whereas texts contained different morphological categories (i.e., articles, nouns, adjectives, adverbs and verbs). This is relevant since it has been shown that the semantic and syntactic properties influence word processing. For example in Italian nouns and adjectives are read faster than verbs [44,45,46].

## Experiment 6: Context Gain and Random Words: Role of Letter Masking and Working Memory in RSVP

In Experiment 5, we examined the effect of context benefits and letter masking by comparing different stimulus types: ordered and scrambled text and unrelated random words. In Experiment 6, to control for the possible confounds described above, all stimuli were taken from the same text, all conditions had the same number and types of morphological categories. Moreover, to maximize masking, we selected random words of the same length (5 letters) so that all the letters in the subsequent word were potentially masked by those in the preceding word.

In the literature a variable number of words was used in each trial. Some studies used a number of targets falling within the working memory span e.g., [19,20] while others used a number of stimuli greater than the span e.g., [16,17,37]. Given the different reading rate results reported in these two sets of studies, we systematically tested the weight of short-term memory in modulating reading rate. To this aim, Experiment 6 was divided into two sub-experiments: In the first, a stream of 6 words was presented in each trial; in the second, each stream consisted of 12 words. In both cases three conditions were run, i.e., ordered text, scrambled text and random words.

### Method

#### Six-word sub-experiment

Six words were presented in each trial. For the ordered text condition, the first 240 words of the first chapter of “*Alice in Wonderland*” were presented in the exact order as they appear in the text (mean word length = 4.9 letters, S.D. = 2.9). For the scrambled text condition, the first 240 words were taken from the second chapter of the book and were randomly presented in the stream of words (mean word length = 4.8 letters, S.D. = 2.8). In the third condition, we selected 240 5-letter words from the whole book. In this condition there were similar percentages of nouns (34.8%), adjectives/adverbs (24.5%) and verbs (40.7%), as in the ordered text (35.4%, 20.8% and 43.7%, respectively) and scrambled text (37.3%, 26% and 36.7%, respectively) conditions.

#### Twelve-word sub-experiment

Twelve words were presented in each trial. For the ordered text condition, the first 480 words of the first chapter of “*Alice in Wonderland*” were presented in the order in which they occur in the text (mean word length = 4.7 letters, S.D. = 2.7). For the scrambled condition, words (the first 480) were taken from the second chapter of the book and were randomly presented in the stream of words (mean word length = 4.8 letters, S.D. = 2.7). In the third condition, we selected 480, 5-letter words from the whole book. Random words included a similar proportion of nouns (37.4%), adjectives/adverbs (23.7%) and verbs (38.7%) as the stimuli used in the ordered (35.5%, 20.9% and 43.6%, respectively) and scrambled text (29.9,% 28.5% and 41.6% respectively) conditions.

Six subjects took part in the first (six-word trial) sub-experiment (mean age = 27 years, range = 23–32; S.D. = 3.9). Six other subjects participated in the second sub-experiment (mean age = 26.8 years, range = 25–32; S.D. = 4.07). The general method described above was followed. No mask was used.

### Results and Comments

**Fig 6:**
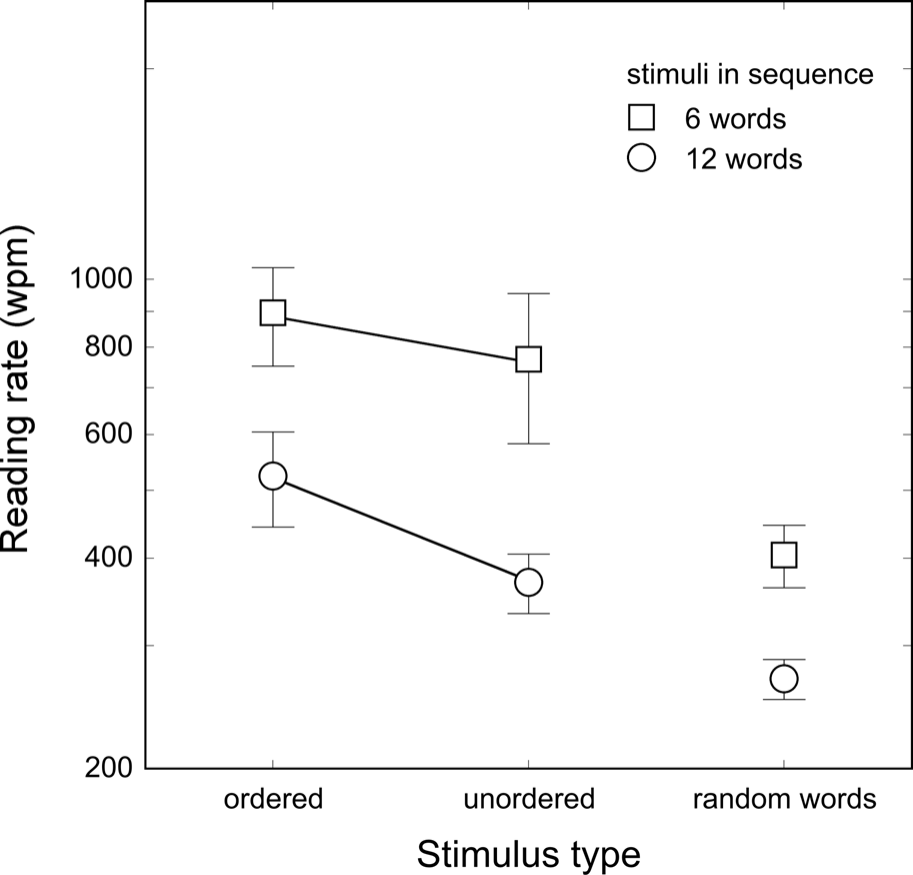
Reading rate (in wpm) for ordered and scrambled text and random (5-letter) words. Two conditions were given: 6-word sequence (black squares) and 12-word sequence (white squares). Bars represent standard deviations.

Results are shown in Fig 6.

For the first sub-experiment (6-word trials), an ANOVA showed that the main effect of stimulus type was significant (F_2, 10_ = 43.06, p < .0001): there was no significant difference between ordered and unordered text conditions (896 and 769 wpm, respectively, p = .12), while both of them were read faster than random words (405 wpm, both ps < .0001). The same analysis was conducted for the second sub-experiment (12-word trials). The main effect of stimulus type was statistically significant (F_2, 10_ = 49.16, p < .0001): a significant context benefit was present with words in ordered text read at a faster rate (524 wpm) than scrambled words (369 wpm, p < .0001). Finally ordered and, most importantly, unordered words were read faster than random words (269 ms; both ps < .0001).

The context benefit, although quantitatively small (a factor of about 1.4), was significant when a large set of words per trial was employed. In the case of a reduced set of words per trial, the context benefit was absent. So, when the stream of targets was within the memory span, context seemed to have a minimal effect on reading rate. The context benefit for the large set of words is presumably linked to the increase in working memory span for semantically related materials [31].

In keeping with the results of Experiment 5, random words of the same length (5 letters in the present experiment) produced the slowest reading rate compared with both ordered and scrambled conditions. The difference between random words and ordered text conditions can in principle be attributed to the absence of a facilitating context for random words, but the significant difference between random words and scrambled text requires a different explanation. In particular, a low processing level interpretation of the data may be advanced. When a stream of words is presented sequentially in the same spatial location and words have the same length, masking between letters of consecutive words reduces the visibility of the letters (and, thus, of the words). Vice versa, when words in the stream have different lengths, as in the scrambled text condition, masking is reduced, favouring the visibility of letters located in non-overlapping positions and appreciably increasing reading rate.

Finally, we tested the role of working memory when reporting words displayed by RSVP by comparing reading rate in the two sub-experiments. A 2 x 3 ANOVA was run with target numerosity (6-, 12-target stimuli) as between-subject factor and stimulus type (ordered text, scrambled text and random words) as repeated factor. Results showed significant main effects of the target numerosity (F_1, 10_ = 11.16, p < .01) and stimulus type (F_2, 20_ = 86.27, p < .0001) factors. The target numerosity by stimulus type interaction was significant (F_2, 20_ = 4.18, p < .05): reading rate for all types of stimuli was higher with fewer items per trial but the advantage was greater for the scrambled and ordered text conditions (p < .01 and .05, respectively) than for the random word (p < .05) condition.

Overall, results indicated that overloading working memory leads to a decline in reading rate by an approximate factor of 2 across conditions, with a somewhat smaller effect for random words. This finding explains some of the inconsistencies found in the literature and will be further examined in the general discussion.

## General Discussion

In RSVP studies, quite different values have been reported as estimates of the reading speed of adult literate readers (i.e. ranging from 100 to 1500 wpm). Here, in six experiments we systematically investigated the variables that modulate reading rate estimates. The results show the critical contributions of factors ranging from low perceptual to high cognitive levels, providing a sort of recipe for comparing rates across labs and targeting different components that contribute to reading rate in RSVP.

Our results indicate that we can read up to 1200 wpm when decoding unmasked words presented in a sequence (Experiment 2). When masks are added at the beginning and the end of the sequence a speed limit of 600 wpm is imposed (Experiments 1 and 3). Notably, words in the stream do not contribute with the same weight to the performance measured in standard RSVP: if no mask is added (as in most studies), the first and last words are more visible and greatly influence overall reading rate. When the first and last word in the stream are unmasked, the presence of context further increases reading rate, although by a small amount (factor of 1.4), and only when the stimuli in the trial exceed the short-term memory span (12 words). Memory imposes a speed limit that ranges between 250 wpm and 500 wpm (Experiments 5 and 6) depending on the load created by the materials used (the speed is lowest for random words and highest for meaningful sentences). Finally, Experiments 4 confirmed the important role played by eye movements and showed their contribution in imposing the upper limit for reading speed, i.e. about 300 wpm.

Below we discuss the factors examined in turn, giving special attention to the masking effect.

### Words order and masking

We found a dramatic effect of masking on the speed of RSVP reading. Visual masking refers to the reduced visibility of a stimulus due to the preceding (forward) or subsequent (backward) presentation of another stimulus in the same spatial location [22,23,24]. Manipulations of the mask characteristics in priming experiments have been used to characterize the nature of the computation involved in word recognition e.g., [47]. In RSVP experiments masking is intrinsic to the paradigm itself. Indeed, in the case of a stream of words, as in the RSVP paradigm without masking, the first and last words are less affected by the masking phenomenon because they are only subjected to backward or forward masking, respectively. Conversely, the central words undergo both backward and forward masking. The addition of a non-verbal mask blunts these effects and smooths the perceptual differences among words in the stream, equalizing their visibility. This is relevant because estimates of reading rate typically take into account the subject’s performance on all words regardless of their ordinal position in the stream.

The presence/absence of the mask drastically affected the estimation of reading rate and contributed to explain the large discrepancies in reading rate found in the RSVP literature. When a mask is used, the median of the reading rate is about 470 wpm [17,18,19,21,32,33,34,35]. Conversely, when no visual mask is used reading rates increase, with a median of about 565 wpm [14,16,20,37,40]. In the present study we show that the first and last word have a large advantage over the other words in the trial, thus confirming the anecdotal observations of Pelli and Tilman (2007) [21]. This advantage is due to reduced feature or letter masking (see below for a discussion). Thus, not all words in the stream have the same visibility. As reading rate is typically measured at about 80% correct, we can conclude that overall RSVP speed is greatly facilitated by the visibility of the first and last words.

### Nature of the masking effect

The main finding of the present study was the in-depth evaluation of masking in the RSVP procedure. What is the nature of this masking effect? We found that a non-orthographic mask (######) had an effect on the preceding item that was quantitatively very similar to that of a word, suggesting that masking occurs mostly at a feature or elementary unit (# and letter) level rather than a word level. These results are in general agreement with repetition experiments in which priming effects may act as early as the feature level with the word shape having limited or no role in lexical access [48,49].

Masking between words in RSVP explains the great advantage of the first and last word. Once we level this advantage by adding the masks before the first and after the last word, we found a small advantage only for the first word in the stream. Different accounts have been proposed to explain the difference among stimuli in the RSVP stream with special reference to attention. Ariga and Yokosawa (2008) [50] demonstrated the existence of an attentional limitation for the detection of a target when it appeared early in the stream. This effect is in the direction opposite to ours. These authors proposed that the deficit with targets that are presented early is due to “attentional awakening” because attentional preparation is required to set up the visual system to detect brief targets; for the following items displayed in a rapid, regular time sequence of events the time of onset is predictable and the “time orientation”, to borrow Rohenkohl and Norbre’s term [51], facilitates perception. Thus, stimuli late in the sequence should receive some degree of facilitation. Ambinder & Lleras (2009) [52] distinguished the attentional awakening mechanism from attentional blink; the latter refers to difficulty in identifying a second target in a stream if it is presented close in time (around 200 ms) to the first target. It is likely that different attentional mechanisms interacted in the present experimental conditions and we can only say which of them dominated.

The differential weight of masking according to word position in the stream seems more consistent with an attentional blink hypothesis. Different theories have been put forward to explain attentional blink. According to the filter theory [53], attentional blink is due to the blocking of input triggered by the appearance of a distracter immediately following the target. As for the attentional awakening theory, this explanation seems suitable for conditions in which an inhibition process is required, e.g., when a target has to be identified among many previous and successive distractors that do not need to be identified. By contrast, this theory does not easily fit the present experimental conditions in which participants were asked to read all the words presented in the stream, thus all stimuli being targets; inhibition was not encouraged. A different attentional blink theory is provided by bottleneck models [54], which postulate that difficulty in processing the stimulus following the target is due to the amount of cognitive processing still devoted to the previously seen target. Consequently, the second target’s perceptual representation cannot be encoded in memory [55,56,57]. If the attentional blink theory can account for the drop in performance at the second and third word, and also at the fourth word in Experiment 3, it does not easily explain the improvement at the last word in the stream found in Experiment 2. It is important to note that the only difference between the two experiments is the presence (Experiment 3) or absence (Experiment 2) of the masks at the beginning and, most relevantly here, at the end of the trial.

Alternatively, reading maybe impaired by the preceding and successive items in the stream because of temporal crowding. This phenomenon refers to the inability to isolate objects in time and it parallels the more widely studied spatial crowding, whereby object identification is compromised by the presence of nearby elements in space [58,59]. Although the interaction between spatial and temporal crowding is still debated several similarities between the two psychophysical phenomena have been reported [60]. Similar to spatial crowding, temporal crowding impairs object identification in strabismus but not anisometropic amblyopia over and above the visual acuity deficit [61,62,63]. Temporal and spatial crowding limit recognition in the normal periphery and can also be measured in the normal fovea [60,64]. Spatial crowding is unlike ordinary masking, sparing detection and showing a different dependency on size, eccentricity and mask contrast [65]. Temporal crowding is unlike forward and backward masking, in that it occurs for long SOAs [60,61,64]. In masking target detection or identification is impaired if the mask appears earlier than 100 ms from the target appearance [22,24,66]. Crowding interference has been shown for SOAs ranging from 200 to 330 ms [60,64]. This timing is consistent with most of our reading rates threshold estimates (from 200 up to 600 wpm). Finally, target flanker similarity greatly affects crowded object identification [67]. In this vein, the slight advantage of the first word in the stream may be due to the weaker temporal crowding exerted by the non-verbal mask compared to the items that are temporally flanked by words.

Therefore, we propose that the dominant mechanism in the condition studied is a low-level perceptual feature or letter integration for both forward and backward effects; however, additional masking at multiple levels of processing [54] as well as time orientation attention [50] and memory [42] factors might contribute with effects in the same or opposite directions depending on the stimulus timing.

### Memory and context effects

This study shows a strong effect of masking in RSVP, which is primarily detectable at low perceptual processing, as discussed above; however, this effect is partially compensated in the case of the first word of the trial (Experiment 3). The size of this compensation is compatible with primacy effects for memory recall e.g., [42,43]. Similarly, low-level perceptual masking eliminates the high-level context advantage (Experiment 5), with context helping reading speed only in the case of a heavy memory load (Experiment 6). Thus, we propose two conclusions: First, low perceptual and high cognitive levels interact when reading rate is measured with RSVP. Second, the advantage due to context is in the foreground only when there are more than six items in the RSVP. These conclusions are consistent with Potter’s view that the RSVP technique is “*capable of revealing very rapid*, *presumably automatic and perhaps elementary cognitive and linguistic operations*, *such as those that structure a string of words into a sentence*” (pg. 97, [68]).

### Role of eye movements

The measurements reported in Experiments 4 confirm the important role of eye movements in limiting reading speed. However, the distance between consecutive words in Experiment 4 was higher than what is typical among words in a text, which may have required artificially larger saccades.

The fact that at face value the rate obtained when random words were displayed along a horizontal line in the present study closely resembles the rate obtained by Carver’s 300 wpm rauding speed [1] deserves some comments. The speed of functional reading (as measured by Carver) was greatly limited by pronunciation time, but pronunciation was not involved in the present RSVP reading. Thus, the similar values recorded cannot be attributed to the same factor. Eye movement execution was forced in the present RSVP of words successively displayed along the horizontal axis, and the contribution of eye movements to reading rate was measured. By contrast, in Carver’s study the reader could skip some words (i.e., he/she might not fixate them) because the text contained short words and function words [69,70,71]; thus, Carver’s rate value should have taken advantage of this skipping. In general, a lower number of eye movements due to skipping should improve reading rate when functional texts are used. Thus, we can speculate that the 300 wpm speed limit [1] may be set by the learned eye-voice coupled pace adopted during reading, as suggested in a previous study [6]. On the other hand, however, Carver’s reported reading times may have also been influenced by regressive saccades which are likely in functional reading [72].

In any case, the present results indicate that eye movement execution have an important role in setting reading speed to 300 wpm even when pronunciation time does not have a relevant role, such as in RSVP reading.

### Concluding remarks

As presented in the Introduction, different previous studies reported very different maximum reading rates but this discrepancy has received little attention. To understand the source of this variability we evaluated the weight, in terms of processing time, of the various components that contribute to the reading process using the RSVP procedure. The present data indicate that various low- and high-level factors exert a significant role on reading rate. The role of each of these factors was isolated and analysed in depth and the present data help clarifying and reconciling the discrepancies among reading rates found in the literature. The crucial effect of eye movements was confirmed, and the much less considered role of masking between successive items was clearly shown. Recent innovation in applied research focused on developing devices and software (like tablets and e-books) that present text according to the speed-reading theory and following the RSVP procedure [73,74]. Such innovative software allows presenting texts in small displays while maintaining good reading time. Many different commercial applications have been developed lately and some of them got very popular (e.g. Spritz; Velocity). The advantage of these applications is that they highlight the optimal recognition point within the word, reducing the need of saccadic eye movements. Although text understanding is beyond the scope of the present paper, is worth noting that comprehension may be the draw back for the usefulness of these applications. In fact, it has been shown that text comprehension is lower in RSVP reading as compared to traditional reading [12,13]. This poorer understanding has been attributed to either the lack of parafoveal pre-processing [12] or to the impossibility of making regressive saccades [13]; see also [75]. Nonetheless, in neuropsychological conditions whereby oculomotor control is drastically compromised (e.g., Balint syndrome, Posterior Cortical Atrophy [PCA]) and visuospatial disorientation causes extreme difficulties in text reading [76], patients may benefit from single-word presentation. Indeed, it has been recently demonstrated that PCA patients’ accuracy is highly enhanced by single word presentation as compared to lines of texts with the individual patient improvement ranging from 6% to 270% [77]. Similarly, individuals with central scotomas read about 40% faster with RSVP as compared to standard presentation [14]. In these cases the limitations associated with the RSVP applications [75] are far outweighed by the benefits.

## Supporting Information

S1 Dataset RSVP thresholds. Participants’ thresholds obtained in the 6 experiments.
(XLS)
